# O008: A multidisciplinary initiative to save antibiotics: the world alliance against antibiotic resistance (WAAR)

**DOI:** 10.1186/2047-2994-2-S1-O8

**Published:** 2013-06-20

**Authors:** J Carlet, C Pulcini

**Affiliations:** 1 ICU, Hopital St Joseph, Paris, France; 2 Infectious diseases, CHU, Nice, France

## Introduction

Background - Bacterial resistance has reached an alarming level worldwide. There is a worrying gap between the current worldwide spread of multiresistant bacteria and the lack of new antimicrobial drugs. Urgent measures are then needed in order to preserve the efficacy of antibiotics.

## Objectives

Our aim was then to raise awareness and call for action to save antibiotics.

## Results

Objectives and results - We created in 2011 an international network, named the World Alliance Against Antibiotic resistance (WAAR). It gathers health professionals, veterinarians, environment specialists, economists, politicians and delegates of the public. This association is supported by 51 professional societies (40 in France and 11 non-French societies) and counts 470 members, from 45 different countries. We have met with government delegates, have communicated on the topic in the media (television, radio, internet, journals...) and in the scientific literature. We have contacted the APUA (Alliance for the Prudent Use of Antibiotics), the BSAC (British Society of Antimicrobial Chemotherapy), React (Action on Antibiotic Resistance) and GARP (Global Association of Risk Professionals) in order to participate in a common international call for action (letter published in November 2012 in the Financial Times). Our next step in the coming months is to suggest to government delegates to conduct a strong initiative showing that antibiotics are a very special class of drugs. Namely, we will suggest that doctors, including those in the outpatient setting, prescribe antibiotics on a dedicated form, mentioning the clinical indication and the duration of therapy. Moreover, a systematic reassessment of therapy around day 3 is of critical importance. We are planning to evaluate these strategies in randomized controlled trials, assessing total antibiotic use as the main outcome. Adding the antibiotics to the UNESCO World Heritage list would also be very useful.

## Conclusion

Conclusion - We believe that such global initiatives are urgently needed worldwide if antibiotics are to be saved. Those precious drugs must be considered as "special" drugs, and actively protected.

## Disclosure of interest

J. Carlet Consultant for Astellas, Other Basilea, C. Pulcini: None declared.

